# Draft Genome Sequences of Two *Arcobacter* Strains Isolated from Human Feces

**DOI:** 10.1128/genomeA.00113-14

**Published:** 2014-04-03

**Authors:** Zaky Adam, Kerri Whiteduck-Léveillée, Michel Cloutier, Wen Chen, Christopher T. Lewis, C. André Lévesque, Edward Topp, David R. Lapen, James T. Tambong, Guylaine Talbot, Izhar U. H. Khan

**Affiliations:** a Eastern Cereal and Oilseed Research Centre (ECORC), Agriculture and Agri-Food Canada, Ottawa, Ontario, Canada; b Southern Crop Protection and Food Research Centre, Agriculture and Agri-Food Canada, London, Ontario, Canada; c Dairy and Swine Research Development Centre, Agriculture and Agri-Food Canada, Sherbrooke, Quebec, Canada

## Abstract

*Arcobacter* species are members of the family *Campylobacteraceae* and are considered emerging enteropathogens and potential zoonotic agents. Here, we report the draft genome sequences of two *Arcobacter* strains isolated from human feces in an effort to provide further genetic resources for understanding the pathogenic dynamics and diversity of this important genus.

## GENOME ANNOUNCEMENT

The genus *Arcobacter* was proposed as a new genus in 1991 by Vandamme et al. (1). This genus currently consists of 18 species that have been isolated from humans and animals as well as water and food sources (2). The first strain in this genus, *Arcobacter butzleri* RM4018, was isolated from a human clinical sample, and its whole genome was sequenced (3). The strain was noted for its ability to grow and survive under diverse environmental conditions, with a large number of annotated proteins associated with respiration, signal transduction, and chemotaxis, as well as DNA repair and adaptation.

We isolated two *Arcobacter* strains (AF1028 and AF1078) from human feces. Based on multiple pairwise alignment of 16S rRNA gene sequences, the strains show 91.5 to 97.5% similarity to other *Arcobacter* species, including *A. nitrofigilis*, *A. butzleri*, *A. skirrowii*, *A. cibarius*, and *A. cryaerophilus*. To gain a better understanding of the genetic and pathogenic mechanisms of these pathogens, we sequenced their whole genomes.

Whole-genome sequencing of the two *Arcobacter* strains was performed using paired-end sequencing reads on an Illumina HiSeq 2500 with TrueSeq V3 chemistry at the National Research Council Canada (Saskatoon, Saskatchewan, Canada). The reads were 101 bp in length and were obtained from 300-bp inserts, with an average coverage of 590×. The quality of the reads was checked with the program FastQC (http://www.bioinformatics.babraham.ac.uk/projects/fastqc/). *De novo* assembly was performed using ABySS version 1.3.6 (4). We used SSPACE version 2.0 (5) to extend and merge the resulting scaffolds based on read-pair information and short overlaps to reduce the number of scaffolds. GapFiller version 1.11 (6) was used to close the gaps between the short scaffolds that are contained within the large scaffolds by replacing the unknown nucleotides (Ns) with true nucleotides based on read-pair information and short overlaps. Mauve Contig Mover version 2.3.1 (7) was applied to order the draft genomes of both strains by using the genomes of *A. butzleri* ED-1 (8) and *A. cibarius* LMG 21996 (9) as reference strains. Consistent with previous reports (8, 9), the genome G+C contents of these strains were relatively low (27%). The genome information for each strain is summarized in Table 1. Gene prediction and annotation were carried out using the RAST annotation server (10). The draft genomes of the two strains have similar, but not identical, numbers of protein-coding sequences. Strain AF1028 contains 2,353 predicted protein-coding sequences (encoding 1,666 functional, 269 proposed functional, and 418 hypothetical proteins), whereas strain AF1078 contains 2,445 predicted protein-coding sequences (encoding 1,671 functional, 277 proposed functional, and 497 hypothetical proteins). Similarly, strain AF1028 contains 64 predicted noncoding RNAs (51 tRNAs, 1 pseudo-tRNA, and 12 rRNAs consisting of 6 copies each of 16S rRNA and 23S rRNA genes), whereas strain AF1078 contains 68 predicted noncoding RNAs (53 tRNAs, 1 pseudo-tRNA, and 14 rRNAs consisting of 7 copies each of 16S rRNA and 23S rRNA genes).

**TABLE 1:** Summary of information for the draft genome sequences of two strains of *Arcobacter* species

Strain	Source	Accession no.	Genome size (bp)	*N*_50_ (bp)	No. of scaffolds (>300 bp)	G+C content (%)
*Arcobacter* sp. AF1028	Human feces	JART00000000	2,414,790	148,259	46	27.24
*Arcobacter* sp. AF1078	Human feces	JARS00000000	2,496,885	127,252	53	27.18

### Nucleotide sequence accession numbers.

The draft genome sequences of the *Arcobacter* strains AF1028 and AF1078 in this study have been deposited as whole-genome shotgun projects at DDBJ/EMBL/GenBank under the accession no. JART00000000 and JARS00000000, respectively. The version of each strain described in this paper is the first version, JART01000000 and JARS01000000, respectively.

## References

[1] Vandamme P Falsen E Rossau R Hoste B Segers P Tytgat R De Ley J 1991 Revision of *Campylobacter*, *Helicobacter*, and *Wolinella* taxonomy: emendation of generic descriptions and proposal of *Arcobacter* gen. nov. Int. J. Syst. Bacteriol. 41:88–103. 10.1099/00207713-41-1-88 1704793

[2] Kayman T Abay S Hizlisoy H Atabay HI Diker KS Aydin F 2012 Emerging pathogen *Arcobacter* spp. in acute gastroenteritis: molecular identification, antibiotic susceptibilities and genotyping of the isolated arcobacters. J. Med. Microbiol. 61:1439–1444. 10.1099/jmm.0.044594-0 22700547

[3] Miller WG Parker CT Rubenfield M Mendz GL Wösten MM Ussery DW Stolz JF Binnewies TT Hallin PF Wang G Malek JA Rogosin A Stanker LH Mandrell RE 2007 The complete genome sequence and analysis of the epsilonproteobacterium *Arcobacter butzleri*. PLoS One 2:e1358. 10.1371/journal.pone.0001358 18159241 PMC2147049

[4] Simpson JT Wong K Jackman SD Schein JE Jones SJ Birol I 2009 ABySS: a parallel assembler for short read sequence data. Genome Res. 19:1117–1123. 10.1101/gr.089532.108 19251739 PMC2694472

[5] Boetzer M Henkel CV Jansen HJ Butler D Pirovano W 2011 Scaffolding pre-assembled contigs using SSPACE. Bioinformatics 27:578–579. 10.1093/bioinformatics/btq683 21149342

[6] Boetzer M Pirovano W 2012 Toward almost closed genomes with GapFiller. Genome Biol. 13:R56. 10.1186/gb-2012-13-6-r56 22731987 PMC3446322

[7] Rissman AI Mau B Biehl BS Darling AE Glasner JD Perna NT 2009 Reordering contigs of draft genomes using the Mauve Aligner. Bioinformatics 25:2071–2073. 10.1093/bioinformatics/btp356 19515959 PMC2723005

[8] Toh H Sharma VK Oshima K Kondo S Hattori M Ward FB Free A Taylor TD 2011 Complete genome sequences of *Arcobacter butzleri* ED-1 and *Arcobacter* sp. strain L, both isolated from a microbial fuel cell. J. Bacteriol. 193:6411–6412. 10.1128/JB.06084-11 22038970 PMC3209197

[9] Adam Z Whiteduck-Leveillee K Cloutier M Chen W Lewis CT Lévesque CA Topp E Lapen DR Tambong JT Talbot G Khan IUH 2014 Draft genome sequence of *Arcobacter cibarius* strain LMG21996^T^, isolated from broiler carcasses. Genome Announc. 2(1):e00034-14. 10.1128/genomeA.00034-14 24558232 PMC3931353

[10] Aziz RK Bartels D Best AA DeJongh M Disz T Edwards RA Formsma K Gerdes S Glass EM Kubal M Meyer F Olsen GJ Olson R Osterman AL Overbeek RA McNeil LK Paarmann D Paczian T Parrello B Pusch GD Reich C Stevens R Vassieva O Vonstein V Wilke A Zagnitko O 2008 The RAST server: Rapid Annotations using Subsystems Technology. BMC Genomics 9:75. 10.1186/1471-2164-9-75 18261238 PMC2265698

